# Can Biomarkers Help to Diagnose Early Heart Failure with Preserved Ejection Fraction?

**DOI:** 10.1155/2015/426045

**Published:** 2015-01-31

**Authors:** Jaroslav Meluzín, Josef Tomandl

**Affiliations:** ^1^Department of Cardiovascular Diseases, St. Anne's Hospital, ICRC, 65691 Brno, Czech Republic; ^2^Department of Cardiovascular Diseases, Masaryk University, Brno, Czech Republic; ^3^ICRC, Brno, Czech Republic; ^4^Department of Biochemistry, Faculty of Medicine, Masaryk University, 62500 Brno, Czech Republic

## Abstract

Early heart failure with preserved ejection fraction (HFpEF) is a frequent disease, but its diagnosis is difficult and relies mostly on the evidence of left ventricular filling pressure (LVFP) elevation during exercise. Several reports have suggested that natriuretic peptides plasma levels reflect exercise-induced increase in LVFP, but they still have significant limitations. In this context, any new laboratory biomarker that can accurately reflect LVFP elevation during exercise is desirable. Recently, cardiotrophin-1, soluble endoglin, ST2, growth differentiation factor 15, galectin-3, and other new laboratory markers associated with LVFP have emerged. However, the current data on the relationship of these biomarkers and diastolic dysfunction are limited to resting conditions. Therefore, their secretion deserves to be tested under the exercise to determine their potential role in making a diagnosis of early HFpEF.

## 1. Introduction

Heart failure with preserved ejection fraction (HFpEF) is associated with high mortality and morbidity [1, 2]. In addition to the presence of typical symptoms and signs of heart failure as well as the finding of nondilated left ventricle with preserved ejection fraction, the pivotal role in establishing a diagnosis of HFpEF has the evidence of left ventricular filling pressure (LVFP) elevation, indicative of a significant diastolic dysfunction [3, 4]. However, in many patients with exertional dyspnea and/or fatigue due to diastolic dysfunction, LVFP and other parameters quantifying diastolic function can be normal under resting conditions. In such patients, exercise is necessary to reveal a diagnosis of HFpEF. Several authors suggested that isolated only exercise-induced HFpEF (recently called early HFpEF) is a frequent disease [5–8]. Borlaug et al. [8] investigated 55 euvolemic patients with exertional dyspnea, left ventricular ejection fraction (LVEF) > 50%, normal brain natriuretic peptide, and normal cardiac filling pressures at rest. The exercise catheterization was used to classify patients as having HFpEF or noncardiac dyspnea. Thirty-two (58%) subjects had exercise-induced pulmonary capillary wedge pressure (PCWP) ≥ 25 mmHg, confirming exercise-induced HFpEF. However, the noninvasive evidence of exercise-induced LVFP elevation is difficult. Several studies that tried to predict exercise LVFP elevation using Doppler echocardiography gave contradictory results [9–14]. Under these conditions, postexercise assessment of plasma levels of biomarkers known to increase in association with an increased myocardial wall stress may represent a new and promising tool to diagnose early HFpEF.

The aim of this review was to discuss the role of biomarkers in establishing a diagnosis of early (i.e., exercise-induced) HFpEF and to summarize the data on the relationship of new biomarkers and LVFP.

## 2. Brief Summary about the Role of Natriuretic Peptides in Establishing a Diagnosis of HFpEF

The clinically most important natriuretic peptides are brain natriuretic peptide (BNP), N-terminal proBNP (NT-proBNP), atrial natriuretic peptide (ANP), and N-terminal proANP (NT-proANP). Natriuretic peptides are synthesized as precursor proteins (preprohormones) that undergo intracellular modification to form prohormones [15]. They are cleaved into amino-terminal segments (N-terminal-proANP or N-terminal-proBNP) and biologically active carboxy-terminal segments (ANP and BNP). Recently, a midregional sequence of proANP (MR-proANP) was successfully clinically utilized [16]. ANP is secreted from atria in normal adult humans and also from the left ventricle in patients with left ventricular (LV) dysfunction [17]. It is released from storage granules in response to atrial stretch [15]. Left atrial pressure seems to be the major stimulus for ANP release during exercise or atrial pacing [18–20]. BNP originates mainly from the left ventricle both in normal adult humans and in patients with LV dysfunction [17, 21] and is synthesized de novo in response to ventricular stretch [15]. The main impulse for the natriuretic peptide release is myocardial stretch (increased wall stress). The association with wall stress creates the link between elevation of intracardiac filling pressures and elevation of natriuretic peptide levels [15]. An increase in BNP in response to elevated LVFP is adaptive and acts to promote natriuresis, diuresis, inhibition of sympathetic nervous activity, and arterial vasodilatation [22]. Plasma half-lives of ANP, BNP, NT-proANP, and NT-proBNP are 1–5 min, 22 min, 60 min, and 120 min, respectively [15, 23]. The plasma level of BNP and of ANP at the peripheral vein had a significant positive correlation with PCWP, LV end-diastolic pressure (LVEDP), LV end-systolic volume index, LV end-diastolic volume index, and a significant negative correlation with cardiac index and LVEF, respectively [17]. Apart from the markers of systolic and diastolic function, there are other factors known to influence natriuretic peptide circulating levels, including tachycardia, history of atrial fibrillation, myocardial ischemia, ventricular pressure overload, treatment with diuretics and angiotensin-converting enzyme inhibitors, age, gender, body mass index, LV hypertrophy, abnormal lung function, creatinine clearance, and patient position at the time of measurement [15, 24–29]. When interpreting serial changes of natriuretic peptides, one has to take into account a considerable intraindividual variability in both BNP and NT-proBNP [30].

Under resting conditions, many researchers studied plasma levels of natriuretic peptides and their diagnostic role in patients with diastolic dysfunction and suspected or proven HFpEF [31–37]. In such patients, secretion of natriuretic peptides from the left ventricle was found to be increased in proportion to the severity of the LV diastolic dysfunction [31, 33]. BNP and NT-proBNP can be used to detect patients with diastolic dysfunction mainly in those having a pseudonormal and restrictive transmitral filling flow pattern during Doppler echocardiography, that is, in subjects with elevated LVFPs. In patients with an abnormal relaxation filling pattern, that is, in those with normal or only mildly elevated LVFPs, the levels of natriuretic peptides can be normal [31, 36]. The limited utility of natriuretic peptides for the detection of milder systolic and diastolic dysfunction under resting conditions was also found in 2024 randomly selected residents of Olmstead County (MN, USA) [38]. In this study, BNP levels shifted upwards as the severity of diastolic dysfunction increased. To make a diagnosis of HFpEF, the combination of natriuretic peptides with clinical and echocardiographic parameters enhances diagnostic accuracy and appears to be a preferable approach [35, 39].

In summary, the ability of natriuretic peptides to diagnose HFpEF results from their capacity to reflect LVFP elevation caused by a significant diastolic dysfunction. When using natriuretic peptides for making a diagnosis of HFpEF, their plasma levels should not be used in isolation from the clinical context and echocardiography, as natriuretic peptides are influenced by many factors other than diastolic function and may give false positive results. In patients with suspected or proven HFpEF, natriuretic peptides are elevated mainly in subjects with advanced diastolic dysfunction but are frequently normal in mild diastolic dysfunction.

## 3. Novel Biomarkers and Determination of Left Ventricular Filling Pressure, Making a Diagnosis of HFpEF

Recently, increasing interest has been given to new agents and substances with the potential to reflect LVFP elevation and thus to contribute to the diagnosis of HFpEF. In this regard, an important attribute of the majority of these biomarkers is their ability to reflect an increase in myocyte stress/stretch. The most promising agents are cardiotrophin-1 [40–44], soluble endoglin [45], pancreatitis-associated protein [46], ST2 [47–49], growth differentiation factor 15 [50, 51], galectin-3 [52–57], and carbohydrate antigen-125 [58], even if one cannot exclude other agents, in which data on their relation to LVFP and HFpEF are still insufficient or missing.

Cardiotrophin-1 (CT-1) is an interleukin 6 related proinflammatory cytokine with a broad spectrum of biological activities, including cardiovascular ones. The release of CT-1 was found to be stimulated by ventricular stretch [40]. The effects of end-diastolic pressure (EDP) elevation were studied on isolated perfused rat hearts [40]. The left ventricle was stretched for 20 min to achieve an EDP 25–30 mmHg from baseline EDP 5-6 mmHg. Ventricular stretch resulted in a prompt and significant rise in perfusate CT-1 and BNP in both Wistar-Kyoto (WKY) and spontaneously hypertensive (SHR) rat hearts. Other authors demonstrated in rat experiments increased expression levels of CT-1 mRNA and protein at the congestive heart failure stage compared with the LV hypertrophy stage and suggested that CT-1 may play a role in ventricular remodeling during transition from LV hypertrophy to heart failure in the rat hypertensive model [41]. López et al. [42] described an association of plasma CT-1 with the progression of heart failure in hypertensive patients. CT-1 was directly (*r* = 0.416, *P* < 0.001) and inversely (*r* = −0.263, *P* < 0.01) correlated with LV mass index and LVEF [42]. In hypertensive patients with heart failure, an excess of myocardial CT-1 protein was found to be associated with LV end-diastolic wall stress and increased collagen type I and type III mRNAs and protein expression in the fibrotic myocardium [43]. Celik et al. [44] studied 57 patients with diastolic heart failure and 33 controls. CT-1 was significantly higher in patients with diastolic heart failure and significantly correlated with NT-proBNP (*r* = 0.349), with the ratio of early diastolic transmitral flow velocity (*E*) to early diastolic velocity of mitral annular motion (*e*′) (*r* = 0.307), and with the estimated mean PCWP (*r* = 0.308). Taken together, all these reports suggest a relationship between CT-1 plasma level and LVFP and heart failure. However, data on the behaviour of CT-1 during exercise are limited to healthy untrained individuals and athletes [59]. The relationship of exercise-induced CT-1 changes in plasma levels and LVFP changes in patients with LV dysfunction is not known.

Endoglin (CD105) is a transforming growth factor-*β* coreceptor that is released into the circulation as soluble endoglin (sEng). Kapur et al. [45] described a significant correlation of sEng with LVEDP (*r* = 0.689), irrespective of LVEF. Using a receiver-operating characteristic curve, sEng levels predicted LVEDP ≥ 16 mmHg with an area under the curve (AUC) of 0.85, exceeding AUC for both atrial (AUC of 0.68) and brain (AUC of 0.65) natriuretic peptide. sEng also decreased in association with a reduced cardiac filling pressure after diuresis.

Pancreatitis-associated protein (PAP) is a cytokine expressed in a wide range of tissues in response to external stress or inflammation. Fitzgibbons et al. [46] showed that PAP levels correlate with the severity of heart failure and are a marker of cardiorenal syndrome, neurohormonal activation, and elevated filling pressures. In addition, PAP is a sensitive and specific marker for increased 6-month heart failure morbidity and 12- and 24-month all-cause mortality.

ST2 is a member of the interleukin 1 receptor family that represents a novel biomarker of mechanical stress measurable in serum [47]. It is linked to cardiac hypertrophy, fibrosis, and ventricular dysfunction. Elevated serum levels of the soluble isoform of ST2 (sST2) are associated with an increased risk of mortality. Bartunek et al. [48] described a significant correlation between serum level of ST2 and LVEDP and B-type natriuretic peptide level. Wang et al. [49] evaluated 107 hypertensive patients with LVEF > 50%. Among them, 68 suffered from HFpEF. AUC for sST2 was 0.80 as compared to 0.70 for NT-proBNP to detect HFpEF. The sST2 concentration was significantly lower in patients with *E*/*e*′ < 8 compared with those with *E*/*e*′ 8–15 or *E*/*e*′ > 15. Multivariate analysis demonstrated that sST2 > 13.5 ng/mL was independently associated with HFpEF. However, Santhanakrishnan et al. [50] did not find a significant difference in ST2 levels between HFpEF patients and controls after adjustment for age, sex, and other clinical covariates.

Increased concentrations of growth differentiation factor 15 (GDF-15) and high-sensitivity troponin T (hsTnT) were found in patients with both HFpEF and heart failure with reduced ejection fraction (HFrEF) as compared to community-based controls [50]. Interestingly, even if regarded as markers of inflammation (GDF-15) and myocyte necrosis (hsTnT), both GDF-15 (*r* = 0.406) and hsTnT (*r* = 0.424) significantly correlated with *E*/*e*′ ratio, a noninvasive surrogate of LVFP. Similarly, both of these biomarkers significantly correlated with wall stress. GDF-15 distinguished HFpEF from controls with an AUC of 0.936. A GDF-15 cut-off value of 879 pg/mL provided 92% sensitivity and 84% specificity. Similar findings concerning GDF-15 were found in a comparison of 142 patients with HFpEF, 86 patients with HFrEF, and 188 healthy elderly controls [51]. As compared to controls, GDF-15 plasma levels were elevated in both HFpEF and HFrEF patients. In HFpEF patients, GDF-15 was associated with the *E*/*e*′ ratio. GDF-15 was at least as good as NT-proBNP for the detection of HFpEF and the combination of both markers was better than NT-proBNP alone.

A novel biomarker in relation to cardiac fibrosis and remodeling that is currently intensively studied is galectin-3 [52]. Its plasma and/or serum levels are increased in acute and chronic heart failure and are linked with worse prognosis [53–56]. In a study of 115 patients with acute dyspnea with and without acutely decompensated heart failure, higher levels of galectin-3 were significantly but weakly correlated with Doppler indices of higher filling pressure (*E*/*e*′, *r* = 0.345) and more extensive diastolic relaxation abnormalities (*e*′, *r* = −0.246) [56]. However, in patients recovering from an acute myocardial infarction with preserved LVEF, plasma levels of galectin-3 did not differ between patients with and without diastolic dysfunction and were not related to PCWP [57].  Table 1 demonstrates correlations of new biomarkers with surrogates of LVFP.

Recently, carbohydrate antigen-125 (CA-125) was found to be helpful in the establishment of diagnosis of HFpEF. Even if the relationship of CA-125 to LV filling pressures was not studied, CA-125 was found to significantly correlate with the maximum left atrial volume, improved diagnostics of HFpEF, and predicted hospitalizations for heart failure [58].

Diagnosis of HFpEF has also been shown to be associated with the elevation of some inflammatory markers such as interleukin 6, interleukin 8, and monocyte chemoattractant protein 1 [63]. However, their problem is a lack of diagnostic specificity. There are several other emerging biomarkers that are or could be associated with a diagnosis and/or prognosis of heart failure, which are discussed in detail elsewhere [64].

In summary, serum or plasma levels of various new biomarkers correlate with the diastolic load. However, limited or no data are available on the reaction of these biomarkers to exercise and on the capacity to utilize such a biomarker reaction to diagnose early HFpEF.

## 4. Response of Natriuretic Peptides to Exercise

In view of a relatively high frequency of HFpEF confined only to exercise, the identification of biomarkers with the ability to promptly react to exercise-induced LVFP elevation is desirable. Because of a relatively low diagnostic specificity of currently used biomarkers, the optimal agent would have the capacity to increase immediately following exercise-induced elevation of LVFP and return to normal baseline value after cessation of exercise and normalization of LVFP. The time coincidence of biomarker and LVFP elevation induced by exercise would provide very powerful evidence that biomarker elevation really reflects the exercise-induced HFpEF. To date, limited data are available on the course of biomarker blood level changes induced by exercise in patients with HFpEF. The majority of reports, published thus far, studied exercise-induced plasma concentrations of BNP and ANP and focused mainly on healthy subjects [60, 65–70], patients with essential hypertension [61, 70, 71] or various cardiac diseases [72], and on subjects with systolic heart failure [21, 73, 74]. Limited data are available concerning the response of natriuretic peptides to exercise in patients with HFpEF.

### 4.1. Response of Natriuretic Peptides to Exercise in Healthy Volunteers

Baker et al. [65] studied plasma levels of N-terminus and C-terminus of the atrial natriuretic propeptide (NT-proANP and ANP) in 12 healthy volunteers before exercise, during exercise at workloads of 25, 50, 75, 100, 125, 150, and 175 W, and at 10, 30, 60, 120, and 240 min after exercise. Both peptides NT-proANP and ANP increased linearly with graded exercise peaking at 10 min after exercise. In contrast to ANP, NT-proANP remained significantly elevated at 30 and 60 min after exercise as compared to preexercise values. Subjects who were able to achieve a larger workload and a higher VO_2_max had in general the ability to increase their circulating concentrations of both peptides NT-proANP and ANP to a greater extent. Circulating concentrations of both peptides had strong positive correlations with systolic and diastolic pressure, heart rate, VO_2_max, and respiratory quotient. The significant correlation of the increase in the plasma concentration of ANP during exercise with the increase in heart rate and systolic blood pressure was confirmed by Saito et al. [66], who also found the association between an increase in plasma ANP concentration and the intensity of a workload. Follenius and Brandenberger [67] studied plasma levels of ANP in 6 normal male subjects. ANP increased rapidly and significantly after 5–10 min of exercise reaching peak levels at the end of the 30 min exercise. During the recovery phase, ANP decreased immediately and had reached control levels within 30 min. Similar findings were found by Somers et al. [68] and Petzl et al. [69]. However, the exercise-induced plasma ANP elevation reached a lesser level as compared to patients with cardiac disorders [69]. Concerning the effect of exercise on circulating BNP concentration, Huang et al. [60] analyzed 138 blood samples from 23 healthy men aged 23 to 27 years. Authors noticed a transient increase in plasma BNP from 3.38 ± 0.50 to 8.21 ± 2.02 pg/mL immediately after exercise. BNP concentration returned to normal levels within 1 h after exercise.

Taken together, exercise results in elevation of natriuretic peptide plasma levels in healthy subjects. The magnitude of this elevation is related to the intensity of exercise. Several factors have been implicated as potential stimuli for the increase of natriuretic peptides plasma levels during exercise. At present, it is unclear whether they act directly on natriuretic peptide elevation or their effects are mediated through the elevation of LVFP during exercise. However, the exercise-induced rise of natriuretic peptides is less than that of patients with cardiac disorders.

### 4.2. Response of Natriuretic Peptides to Exercise in Patients with Various Cardiac Diseases

Steele et al. [73] investigated circulating plasma levels of ANP and BNP in 10 patients with stable chronic heart failure with LVEF ≤ 40% and in 10 normal control subjects. Levels of ANP and BNP were higher at both rest and peak exercises in patients with heart failure. The rise in ANP at peak exercise was significant in patients compared with the resting level, but not in controls. For BNP, there was a significant rise in patients but no change in control subjects. The circulating plasma levels of both peptides showed a strong negative correlation with LVEF. Matsumoto et al. [21] showed in 7 patients with congestive heart failure due to dilated cardiomyopathy that a symptom-limited exercise test resulted in a significant increase in plasma levels of both ANP and BNP. Keller et al. [18] investigated 33 patients with congestive heart failure using right-sided heart catheterization during supine graded bicycle exercise. Plasma ANP concentrations were elevated at rest and rose considerably during exercise. Of the functional and hemodynamic variables, including right atrial pressure, pulmonary arterial pressure, PCWP, systolic and diastolic blood pressure, plasma ANP correlated most strongly with PCWP both at rest and during exercise. Wijbenga et al. [74] analyzed plasma concentrations of natriuretic peptides at rest and at peak exercise in 52 patients with chronic systolic heart failure. They found clinically significant differences in response to exercise between individual peptides. The percentage exercise-induced increases in ANP, BNP, NT-proBNP, and NT-proANP were 59 ± 58%, 38 ± 52%, 24 ± 24%, and 5 ± 18%, respectively. In 12 patients with old myocardial infarction, Matsubara et al. [19] described a significant correlation (*r* = 0.7, *P* < 0.05) of exercise-induced changes of ANP levels (ΔANP) and exercise-induced changes of PCWP. No significant correlations existed between ΔANP and exercise-induced changes of heart rate, mean blood pressure, pulmonary artery diastolic pressure, cardiac index, and plasma norepinephrine or epinephrine levels, respectively. These findings suggest that ANP secretion is primarily stimulated by the increased atrial pressure.

Kohno et al. [61] studied 6 patients with essential hypertension. They underwent right heart catheterization with graded exercise on a supine bicycle ergometer. The mean pulmonary arterial BNP concentration at rest was 14.8 ± 4.1 pg/mL and increased gradually during exercise, reaching 40.9 ± 6.5 pg/mL at the maximum exercise stage. Close correlations of pulmonary arterial pressure (*r* = 0.83) and pulmonary arterial wedge pressure (*r* = 0.82) with pulmonary arterial BNP concentration were observed. These data suggest that the wall stress caused by exercise stimulates secretion of BNP from ventricles in hypertensive patients. Nishikimi et al. [70] investigated the effect of exercise on plasma concentrations of adrenomedullin, BNP, and ANP in 10 normotensive subjects and in 15 patients with essential hypertension. Plasma levels of all three peptides at rest were significantly higher in hypertensive than in control patients. Plasma concentrations of ANP increased with exercise in both groups and had greater increments in hypertensive patients. Plasma concentrations of BNP increased with exercise only in patients with hypertension. Plasma adrenomedullin did not change with exercise in either group. Tanaka et al. [71] described the exercise-induced increase in BNP as well as ANP, accompanied by an increase in blood pressure, heart rate, and plasma norepinephrine or epinephrine in both normal subjects and hypertensive patients. Univariate and multivariate regression analyses demonstrated that in the normal subjects the exercise-induced release of BNP and ANP was mediated by plasma epinephrine or norepinephrine, respectively, whereas heart rate mediated release of BNP and ANP in the hypertensive patients.

In summary, in patients with cardiac diseases, the exercise-induced increases in natriuretic peptides exceed those found in healthy controls. There exists a significant correlation between natriuretic peptide levels and LVFP during exercise. There are significant differences in response to exercise between individual natriuretic peptides.

### 4.3. Response of Natriuretic Peptides to Exercise in Patients with Suspected or Proven HFpEF

Mottram et al. [62] analyzed results of 26 hypertensive patients with suspected diastolic heart failure (exertional dyspnea, diastolic dysfunction, and LVEF > 50%) at rest and during exercise. Peak exercise BNP was higher in subjects with elevated LVFPs at peak exercise (*E*/*e*′ > 10) compared to those with normal filling pressures (123 ± 124 versus 45 ± 71 pg/mL, *P* = 0.027).  Table 2 demonstrates reactions of BNP levels to exercise in healthy controls, in patients with hypertension, and in subjects with hypertension and suspected or proven HFpEF. Even if a direct comparison among the studies presented cannot be made, there appears a clear trend towards the elevation of exercise BNP levels with progressive worsening of diastolic function. Tschöpe et al. [75] studied 15 controls and 15 patients with HFpEF. In subjects with normal resting PCWP (<12 mmHg) and LVEDP (<16 mmHg), there was only a weak correlation of log NT-proBNP with PCWP at rest (*r* = 0.37, *P* = 0.051) and LVEDP at rest (*r* = 0.39, *P* = 0.044). However, log NT-proBNP was strongly associated with PCWP at exercise (*r* = 0.78, *P* < 0.001) when PCWP was elevated in patients with HFpEF in the range of 20–40 mmHg. The same authors [33] demonstrated in a larger population of 68 patients with HFpEF that NT-proBNP levels obtained at rest may correlate better with LVFPs measured at peak exercise than with those measured at rest. Similar results were described by Fukuta and Little [76] in 80 patients with an impaired relaxation pattern of Doppler LV filling. While there was no significant correlation between the resting *E*/*e*′ ratio and BNP (*r* = 0.18), the *E*/*e*′ obtained immediately after exercise correlated significantly with BNP (*r* = 0.56). Recently, Andersen et al. [57] found in patients recovering from an acute myocardial infarction with preserved LVEF a weak but significant correlation between MR-proANP and PCWP at rest (*r* = 0.33, *P* = 0.002) and at peak exercise (*r* = 0.35, *P* = 0.002). MR-proANP was collected at rest before the exercise.

Taken together, results of the above presented studies suggest that increasing LVFP during exercise is an important trigger for natriuretic peptide secretion. In patients with suspected or proven HFpEF, natriuretic peptide plasma levels may be helpful in the identification of an exercise-induced increase in LV filling pressures.

## 5. Conclusion

The diagnosis of early HFpEF relies most frequently on the evidence of LVFP elevation during exercise. However, noninvasive echocardiographic detection of exercise-induced LVFP elevation is difficult and the results are controversial. In this context, a serologic parameter that reflects LVFP elevation during exercise would be clinically very useful. Numerous reports suggest that natriuretic peptides have the potential to improve the establishment of diagnosis of early HFpEF but still have limitations. Recently, CT-1, sEng, GDF-15, ST2, galectin-3, and other new cardiac markers associated with LVFP have emerged. However, the current data on the relationship of these biomarkers and diastolic dysfunction are limited to resting conditions. Therefore, their secretion deserves to be tested under exercise to determine their potential role in the diagnosis of early HFpEF.

## Figures and Tables

**Table 1:** Correlations of new biomarkers with surrogates of left ventricular filling pressure.

Reference	*n*	Biomarker	Surrogates of LVFP			*P*
			*E*/*e*′	PCWP	LVEDP	
Celik et al. [44]	57	CT-1	0.307^*^			0.003

Kapur et al. [45]	82	sEng			0.689^*^	<0.0001

Bartunek et al. [48]	121	ST2			0.37^*^	<0.01

Santhanakrishnan et al. [50]	151	ST2	0.256^*^			0.002
		GDF-15	0.406^*^			<0.001
		hsTnT	0.424^*^			<0.001

Shah et al. [56]	115	Galectin-3	0.35^*^			0.01

Andersen et al. [57]	74	Galectin-3		NNI		NS

CT-1: cardiotrophin-1; E: early diastolic transmitral flow velocity; *e*′: early diastolic velocity of mitral annular motion; GDF-15: growth differentiation factor 15; hsTnT: high-sensitivity troponin T; LVEDP: left ventricular end-diastolic pressure; LVFP: left ventricular filling pressure; PCWP: pulmonary capillary wedge pressure; sEng: soluble endoglin; NNI: number not included in the paper; NS: not significant; ^*^correlation coefficients are included.

**Table 2:** Responses of BNP levels to exercise in healthy volunteers, patients with hypertension, and in subjects with hypertension and suspected or proven HFpEF.

Reference	*n*	Diagnosis	Biomarker	Plasma levels		*P*
				Rest	Exercise	
Huang et al. [60]	23	Healthy cont.	BNP (pg/mL)	3.38 ± 0.50^*^	8.21 ± 2.02^*^	<0.01

Kohno et al. [61]	6	Hypertension	BNP (pg/mL)^**^	14.8 ± 4.1^**^	40.9 ± 6.5^**^	<0.01

Mottram et al. [62]	26	Hypertension + HFpEF sp	BNP (pg/mL)	48 ± 57	74 ± 97	<0.001
					123 ± 124^***^	

Results are presented as mean ± SD unless otherwise stated. Cont.: controls; sp: suspected or proven; ^*^mean ± SEM; ^**^pulmonary arterial BNP levels; ^***^in a subgroup of patients with elevated left ventricular filling pressure.
